# Short-term hyperoxia does not exert immunologic effects during experimental murine and human endotoxemia

**DOI:** 10.1038/srep17441

**Published:** 2015-11-30

**Authors:** Dorien Kiers, Jelle Gerretsen, Emmy Janssen, Aaron John, R. Groeneveld, Johannes G. van der Hoeven, Gert-Jan Scheffer, Peter Pickkers, Matthijs Kox

**Affiliations:** 1 Department of Intensive Care Medicine, Radboud university medical center, Geert Grooteplein Zuid 10, Nijmegen, 6500 HB, Netherlands; 2 Department of Anesthesiology, Radboud university medical center, Geert Grooteplein Zuid 10, Nijmegen, 6500 HB, Netherlands; 3 Radboud Centre for Infectious Diseases (RCI) Geert Grooteplein Zuid 10 PO Box 9101, 6500 HB Nijmegen, The Netherlands

## Abstract

Oxygen therapy to maintain tissue oxygenation is one of the cornerstones of critical care. Therefore, hyperoxia is often encountered in critically ill patients. Epidemiologic studies have demonstrated that hyperoxia may affect outcome, although mechanisms are unclear. Immunologic effects might be involved, as hyperoxia was shown to attenuate inflammation and organ damage in preclinical models. However, it remains unclear whether these observations can be ascribed to direct immunosuppressive effects of hyperoxia or to preserved tissue oxygenation. In contrast to these putative anti-inflammatory effects, hyperoxia may elicit an inflammatory response and organ damage in itself, known as oxygen toxicity. Here, we demonstrate that, in the absence of systemic inflammation, short-term hyperoxia (100% O_2_ for 2.5 hours in mice and 3.5 hours in humans) does not result in increased levels of inflammatory cytokines in both mice and healthy volunteers. Furthermore, we show that, compared with room air, hyperoxia does not affect the systemic inflammatory response elicited by administration of bacterial endotoxin in mice and man. Finally, neutrophil phagocytosis and ROS generation are unaffected by short-term hyperoxia. Our results indicate that hyperoxia does not exert direct anti-inflammatory effects and temper expectations of using it as an immunomodulatory treatment strategy.

In critically ill patients, the treatment paradigm “treat first what kills first” emphasizes on the avoidance of hypoxia and liberal oxygen supply is often the first medical intervention to be initiated, frequently resulting in hyperoxia at ICU admission^1^,^2^,^3^,^4^. A recent meta-analysis of observational studies revealed an association between hyperoxia at ICU-admission and increased mortality, albeit this was mainly due to increased mortality in a large subgroup of patients with cardiac arrest^5^. On the contrary, hyperoxia might also exert beneficial effects, for instance as prophylactic treatment for surgical wound infections, although clinical trials have yielded conflicting results^6^. The mechanism through which hyperoxia might exert detrimental or beneficial effects and contributes to outcome in critically ill patients is largely unclear, but immunologic effects might play a role. *In vitro,* short-term hyperoxia was shown to attenuate cytokine production^7^, β2-integrin expression necessary for leukocyte adhesion^8^, and macrophage phagocytosis and killing^9^. Furthermore, animal studies have demonstrated that hyperoxia mitigates the inflammatory response and organ damage after administration of zymosan^10^ and cecal ligation and puncture (CLP)^11^,^12^. However, these beneficial effects of hyperoxia *in vivo* were found 20-48 hours after the inflammatory insult^10^,^11^,^12^. As such, it remains unclear whether these were due to direct immunosuppressive effects of hyperoxia, or resulted from preserved tissue oxygenation during severe hemodynamic instability, thereby preventing additional tissue damage and subsequent inflammation^13^,^14^.

If hyperoxia has intrinsic anti-inflammatory effects, it could be a promising treatment option in inflammatory conditions in the ICU, as oxygen is affordable and widely available. However, evidence of direct immunologic effects of hyperoxia in animals and humans *in vivo* is lacking. In addition, there are concerns of oxygen toxicity in the lungs, characterized by a pulmonary inflammatory response and lung injury^15^,^16^.

In the present study, we investigated the intrinsic immunologic effects of short-term hyperoxia in the presence and absence of systemic inflammation elicited by administration of LPS in mice and man, primarily reflected by circulating cytokine levels. To evaluate possible compartmentalization of immunologic effects of hyperoxia, we also determined cytokine concentrations in spleen, liver, and lung homogenates in mice. Furthermore, as hyperoxia has been reported to impair leukocyte functions (e.g. cytokine production^7^, phagocytosis and killing^9^), whole blood ex vivo cytokine production, neutrophil phagocytosis, and intracellular generation of reactive oxygen species (ROS) were assessed in humans.

## Results

### Effects of hyperoxia during murine endotoxemia

**Figure 1:**
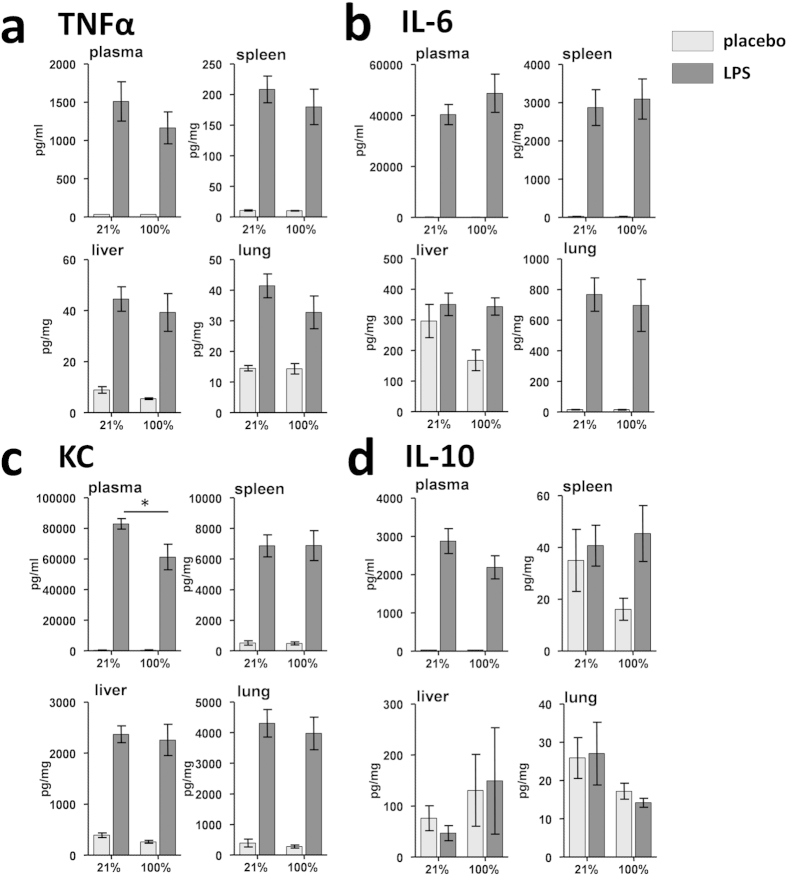
Cytokine concentrations in different compartments in mice. Plasma, spleen, liver, and lung concentrations of (**a**) TNF-α, (**b**) IL-6, (**c**) KC, and (**d**) IL-10 150 minutes after normoxia/hyperoxia (90 minutes after LPS/placebo administration). Concentrations are represented as mean ± SEM. *indicates p < 0.05.

Hyperoxia was well tolerated and did not increase cytokine levels in plasma or tissue homogenates in placebo-treated mice (Fig. 1). LPS administration resulted in increased cytokine levels in tissue homogenates, with the exception of IL-6 in liver, and IL-10 in spleen, liver, and lung homogenates. Apart from a slight, but statistically significant, reduction in plasma KC, hyperoxia did not affect LPS-induced cytokine concentrations.

### Effects of hyperoxia during experimental human endotoxemia

#### Demographic characteristics and safety

Demographic characteristics of the subjects are listed in Table 1 and were similar among the groups. Hyperoxia was well tolerated. No (serious) adverse events occurred during the study.

#### Oxygenation, hemodynamic parameters, temperature, and symptoms

**Figure 2:**
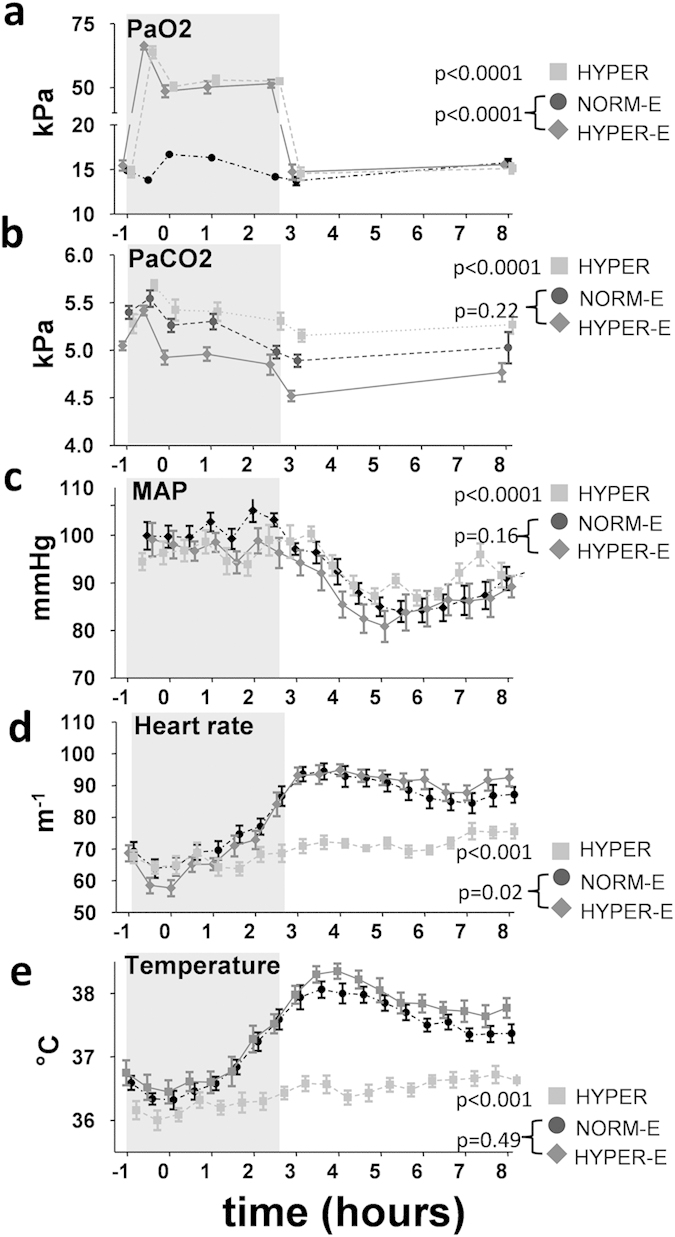
Blood gas parameters, hemodynamics, and temperature in healthy volunteers. (**a**) arterial oxygen pressure (PaO_2_), (**b**) arterial carbon dioxide pressure (PaCO_2_), (**c**) mean arterial pressure (MAP), (**d**) heart rate, and (**e**) tympanic temperature. The period of hyperoxia or normoxia is indicated with a grey box. In the endotoxemia groups, LPS was administered at T = 0 hours. Data are expressed as mean ± SEM. NORM-E: normoxic endotoxemia, HYPER-E: hyperoxic endotoxemia, HYPER: hyperoxia.

During the hyperoxic/normoxic period, mean PaO_2_ was similar in the hyperoxia group and hyperoxic endotoxemia group (54.8 ± 3.0 (411.7 ± 22.8) and 54.1 ± 4.1 (405.9 ± 31.1) kPa (mmHg), p = 0.89), whereas PaO_2_ in the hyperoxic endotoxemia group was higher than in the normoxic endotoxemia group (15.2 ± 0.7 (114.3 ± 5.5) kPa, p < 0.0001, Fig. 2a). PaCO_2_ was 5.0 ± 0.3 (37.8 ± 1.9) kPa in the hyperoxia group, 5.5 ± 0.2 (40.9 ± 1.2) kPa in the hyperoxic endotoxemia group, and 5.3 ± 0.2 (39.5 ± 1.7) kPa in the normoxic endotoxemia group (Fig. 2b). After an initial increase, PaCO_2_ decreased over time in the hyperoxia group. A similar pattern was observed in the normoxic endotoxemia and hyperoxic endotoxemia groups, with no differences between these groups (Fig. 2b). Lactate levels were in the normal range (<2 mmol/L) throughout the experiment in all subjects. In all groups, mean arterial pressure (MAP) decreased over time, with no difference between the normoxic and hyperoxic endotoxemia groups (Fig. 2c).

In the hyperoxia group, the heart rate gradually increased during the day, while a pronounced increase was observed in both endotoxemic groups (Fig. 2d). Endotoxemia resulted in a typical increase in temperature of approximately 1.5 °C (Fig. 2e), and flu-like symptoms (median [IQR] peak symptom scores 1.5 h after LPS administration of 5.9 [0.81] and 8.7 [1.7] in the normoxic and hyperoxic endotoxemia groups, p = 0.20), both of which were not influenced by hyperoxia.

#### Plasma *c*ytokines

**Figure 3:**
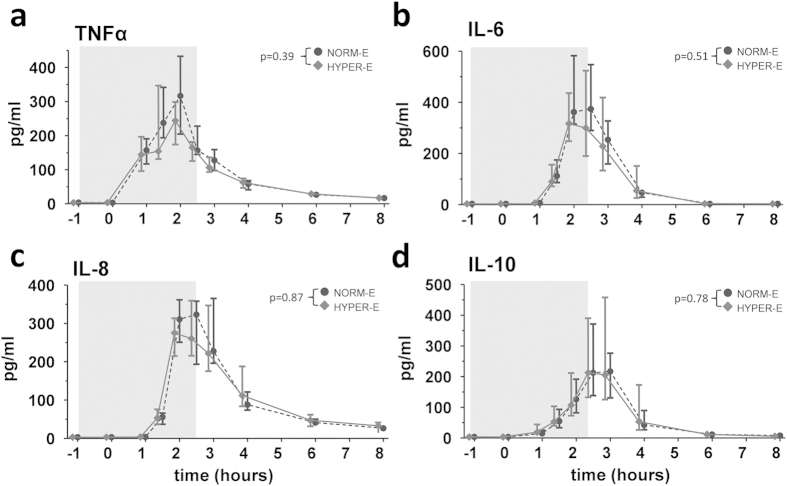
Plasma cytokine concentrations in healthy volunteers. Plasma concentrations of (**a**) tumor necrosis factor (TNF)α, (**b**) interleukin (IL)-6, (**c**) IL-8 and (**d**) IL-10. The period that subjects were fitted with the respiratory helmet and breathed hyperoxic or normoxic air is indicated with a grey box. In the endotoxemia groups, LPS was administered at T = 0 hours. Data are expressed as median with interquartile range. NORM-E: normoxic endotoxemia, HYPER-E: hyperoxic endotoxemia, HYPER: hyperoxia.

Plasma cytokine concentrations were below the lower detection limit of the assay at all time-points in the hyperoxia group. LPS administration resulted in increased plasma concentrations of TNFα, IL-6, IL-8, and IL-10 in all subjects. No differences between the normoxic and hyperoxic endotoxemia groups were observed (Fig. 3).

#### Circulating leukocytes

**Figure 4:**
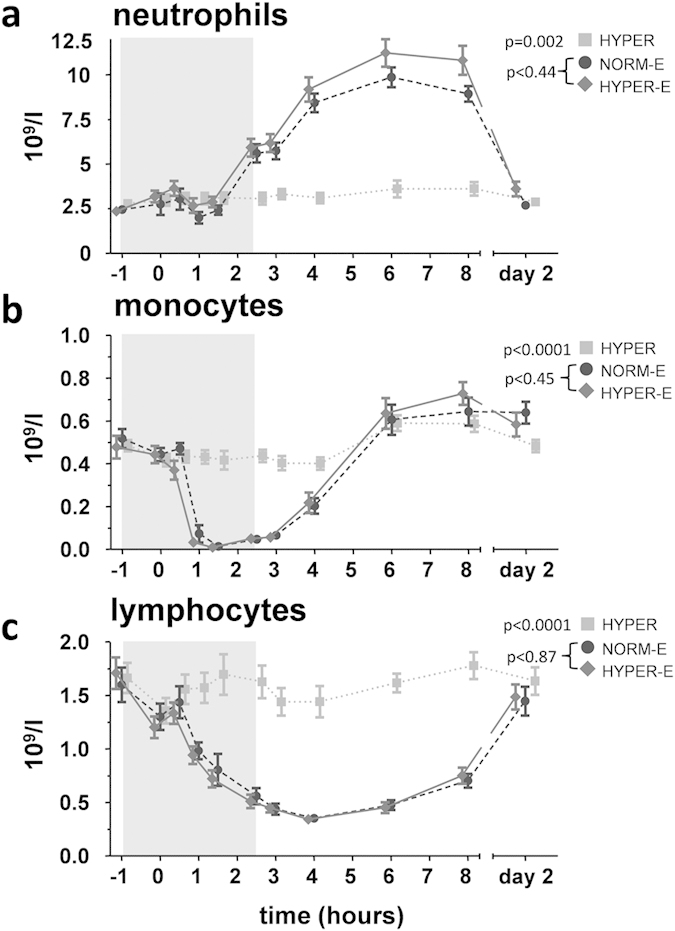
Circulating leukocytes in healthy volunteers. Numbers of circulating (**a**) neutrophils, (**b**) monocytes and (**c**) lymphocytes. The period that subjects were fitted with the respiratory helmet and breathed hyperoxic or normoxic air is indicated with a grey box. In the endotoxemia groups, LPS was administered at T = 0 hours. Data are expressed as mean ± SEM NORM-E: normoxic endotoxemia, HYPER-E: hyperoxic endotoxemia, HYPER: hyperoxia.

Numbers of circulating neutrophils, monocytes, and lymphocytes changed significantly, but to no clinically relevant extent, over time in the hyperoxia group (Fig. 4). LPS administration typically resulted in neutrophilia, with transient lympho- and monocytopenia. Hyperoxia did not affect LPS-induced changes in any of the leukocyte subsets.

#### Ex vivo cytokine production

**Figure 5:**
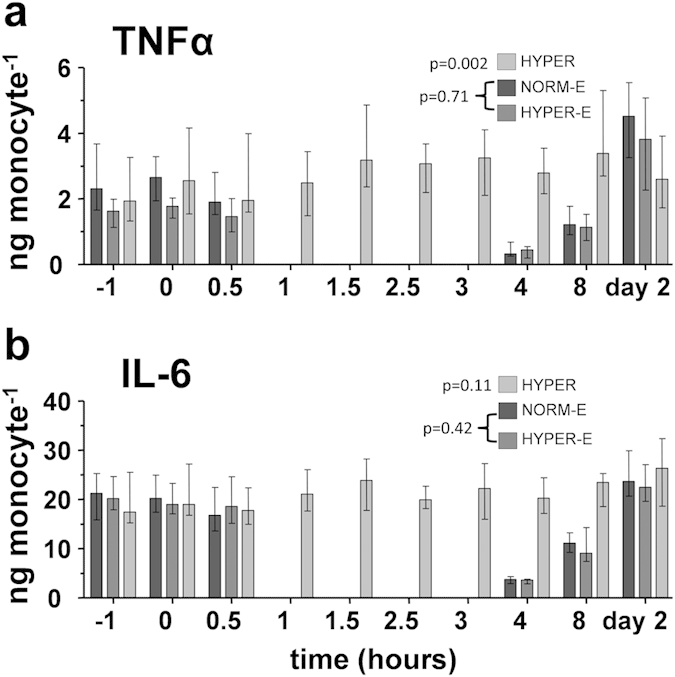
*Ex vivo* whole blood stimulation in healthy volunteers. (**a**) Tumor necrosis factor (TNF)α and (**b**) interleukin (IL)-6 cytokine production in whole blood stimulated *ex vivo* with LPS. Data are expressed as median with interquartile range. NORM-E: normoxic endotoxemia, HYPER-E: hyperoxic endotoxemia, HYPER: hyperoxia.

In the hyperoxia group, *ex vivo* production of TNFα was slightly increased at several time-points, but no clear relationship with the period of hyperoxia was evident (Fig. 5a). Furthermore, IL-6 production was unaffected (Fig. 5b). As circulating monocytes decrease during endotoxemia, ex vivo cytokine production could not be assessed one to three hours after LPS administration. As expected, in both endotoxemic groups leukocyte cytokine production capacity was blunted four and eight hours after LPS-administration due to endotoxin tolerance, which fully recovered the subsequent day. There were no differences in *ex vivo* cytokine production between the normoxic and hyperoxic endotoxemia groups.

#### Neutrophil phagocytosis and ROS generation

**Figure 6:**
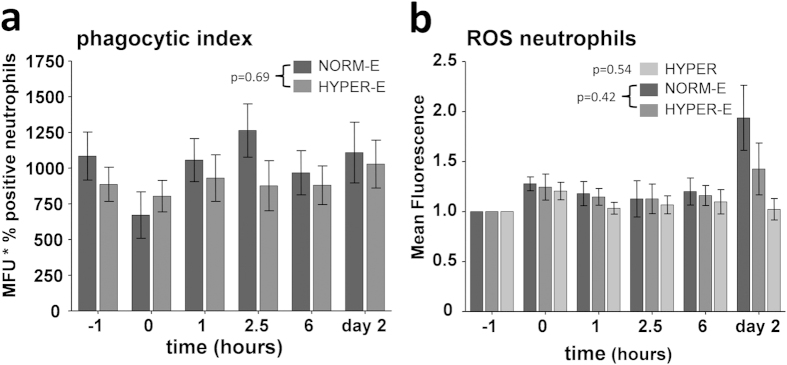
Neutrophil phagocytosis and ROS generation in healthy volunteers. (**a**) Neutrophil phagocytic index and (**b**) neutrophil intracellular ROS generation. Data are expressed as mean ± SEM. NORM-E: normoxic endotoxemia, HYPER-E: hyperoxic endotoxemia, HYPER: hyperoxia.

Neutrophil phagocytosis was similar in the normoxic and hyperoxic endotoxemia groups, with no clear changes over time following LPS administration (Fig. 6a). Intracellular ROS generation was not affected in the hyperoxia group at any time-point. Also, no differences were observed between both endotoxemic groups (Fig. 6b).

## Discussion

In the present study, we demonstrate that short-term hyperoxia neither induces increased levels of cytokines in itself, nor modulates the cytokine response during systemic inflammation induced by endotoxin administration in mice and healthy volunteers. In addition, both in the presence and absence of systemic inflammation in humans, no relevant effects of short-term hyperoxia were observed on temperature, flu-like symptoms, leukocyte counts, ex vivo whole blood cytokine production, neutrophil phagocytosis, and neutrophil ROS generation. To the best of our knowledge, we are the first to investigate the intrinsic immunomodulatory effects of hyperoxia in a standardized and well-controlled model of systemic inflammation in humans *in vivo*.

Our findings appear to be in contrast with previous animal studies reporting anti-inflammatory effects of hyperoxia during systemic inflammation. Although exposure to 100% oxygen for 20 hours or to 70% oxygen for 48 hours did not affect plasma TNFα levels after CLP in rats, intermittent exposure to 100% oxygen for 6 hours per day reduced plasma TNFα concentrations^12^. Similarly, intermittent hyperoxia for 2–3 hours attenuated plasma TNFα and IL-6 levels 24 hours after zymosan administration in mice^10^. Paradoxically, the same authors showed that both shorter and longer exposure to 100% oxygen nullified the anti-inflammatory effects, without a clear explanation^10^. The discrepant results between these studies and ours might be explained by the use of more prolonged and severe models of inflammation. Prolonged inflammation may lead to a vicious cycle by causing tissue hypoxia, which subsequently results in the release of danger associated molecular patterns (DAMPs), in turn leading to aggravation of the inflammatory response^17^. Along these lines, in animal models of severe sepsis and septic shock, hyperoxia was shown to improve tissue oxygenation and organ function, ultimately resulting in better survival^18^,^19^. Therefore, the previously observed beneficial effects of hyperoxia on systemic inflammation might be attributable to the prevention of tissue hypoxia and therefore interruption of the vicious cycle, rather than intrinsic immunosuppressive effects. The short-term murine endotoxemia model used in the present study is very unlikely to have resulted in tissue hypoxia, and the human endotoxemia model is naturally too mild to cause significant hemodynamic instability and tissue hypoxia.

Human *in vivo* studies on the effects of hyperoxia on the inflammatory response are scarce, and have mainly been performed in the perioperative setting. In a trial in patients undergoing elective thyroid surgery, a perioperative FiO_2_ of 80% resulted in decreased levels of postoperative plasma C-reactive protein, IL-1β, and IL-6 compared with a FiO_2_ of 30%. The authors suggest that hyperoxia prevented wound hypoxia and consequently attenuated inflammation^20^. In contrast, hyperoxic ventilation in coronary artery bypass graft surgery resulted in a small, but significant, increase in plasma IL-6 on the first postoperative day^21^. However, this could not be reproduced in an identical study performed by the same group, where hyperoxia did not affect postoperative plasma concentrations of IL-6, IL-8, TNFα, IL-1a, IL-1β, and IL-10^22^. A number of randomized clinical trials have been conducted investigating perioperative hyperoxia as a means to prevent surgical site infections. Meta-analyses of these studies report a slight beneficial effect in e.g. colorectal surgery^6^,^23^.

Although *in vitro,* hyperoxia has been shown to enhance LPS-induced whole blood cytokine production^7^, we now demonstrate that hyperoxia *in vivo* does not affect *ex vivo* whole blood cytokine responses. As such, the present findings suggest that hyperoxia *in vivo* does not result in (lasting) leukocyte reprogramming that modifies cytokine production capacity. Previous studies have also described that hyperoxia inhibits phagocytosis of alveolar macrophages *in vitro*^9^ and *in vivo* in mice^24^, whereas neutrophil phagocytosis and ROS generation was shown to be enhanced *in vitro*^25^. In contrast, *ex vivo* neutrophil ROS generation and phagocytosis remained unaffected after exposure of healthy volunteers to hyperbaric hyperoxia^26^, which is in agreement with our findings. Taken together, these results suggest that the effects of hyperoxia on phagocytosis and killing is highly cell-type specific.

The absence of immunomodulatory effects of hyperoxia in the present study tempers expectations of utilizing therapeutic hyperoxia in acute inflammation. In addition, the concept of therapeutic hyperoxia requires cautious consideration for several other reasons.

First, an important drawback is the risk of oxygen toxicity^27^. The lungs, being the gas exchange interface, are at the greatest risk of hyperoxia-induced injury^15^. Pulmonary oxygen toxicity ultimately resembles the clinical and pathological characteristics of ARDS^27^, as demonstrated in mice exposed to 100% oxygen for up to 48 hours^16^. These mice developed neutrophil infiltration and increased IL-6 and TNFα levels in the bronchial alveolar lavage fluid within 12 hours, followed by alveolar congestion, wall thickening, and hemorrhage^16^. Other animal studies have shown that especially in acute lung injury, high FiO_2_ levels are detrimental as well^28^. The current study was not primarily designed to assess oxygen toxicity, as this has only been described after long-term hyperoxia^24^,^28^,^29^. Nevertheless, we demonstrate that short-term hyperoxia neither causes pulmonary inflammation in itself, nor does it aggravate the pulmonary inflammatory response induced by systemic LPS administration in mice.

A second concern is related to possible hemodynamic effects of hyperoxia, as it was shown to induce vasoconstriction and hence reduced tissue blood flow^30^,^31^ as well as reduced coronary blood flow and myocardial oxygen consumption^32^ in humans. Although we did not observe an effect of hyperoxia on gross hemodynamic parameters during human endotoxemia, we did not assess regional blood flow, oxygen consumption, or vascular resistance. Currently, the net effects of hyperoxia on hemodynamic parameters and oxygen delivery in patients with sepsis are unknown.

Finally, hyperoxia at ICU admission was shown to be associated with increased mortality in a meta-analysis of observational cohort studies^5^, which might be related to the issues raised in the previous points. Of note, this overall effect might be attributed to a large subgroup of cardiac arrest patients in the meta-analysis, in whom hyperoxia has obvious detrimental effects. Naturally, physicians may be tempted to treat the most severely ill patients with a higher FiO_2_, therefore, hyperoxia could represent a confounding factor instead of a direct cause of increased mortality in these observational studies.

This study has several strengths. First, results obtained in animals were confirmed in a human *in vivo* model of systemic inflammation. This is important, as the acute inflammatory response in human and animal models may differ considerably^33^. Second, it is unlikely that tissue oxygenation was compromised in our experiments. Therefore, conclusions can be drawn about the intrinsic effect of hyperoxia on the inflammatory response, without interference of tissue hypoxia effects or (other) alterations that might emerge during long periods of hyperoxia. Nevertheless, endotoxemia models do not reflect all aspects of the complex systemic inflammatory response encountered in many critically ill patients. Therefore, the effects of oxygenation on outcome in systemically inflamed ICU patients remain to be determined. Data from a recently preliminary terminated clinical trial in which sepsis patients were randomized to either hyperoxia or normoxia and hypertonic or isotonic saline in a 2 × 2 factorial design (HYPER2S, ClinicalTrials.gov identifier NCT01722422), and an ongoing study on liberal versus restrictive oxygenation in ICU patients with systemic inflammation (O2-ICU ClinicalTrials.gov identifier NCT02321072) will hopefully answer this important question.

In conclusion, short-term hyperoxia does not affect the inflammatory response induced by administration of endotoxin in mice and man. These results indicate that hyperoxia does not exert direct anti-inflammatory effects, and suggest that the previously reported beneficial effects in animal models of more prolonged and severe inflammation are due to preserved tissue oxygenation. Our data tempers expectations of using hyperoxia as an immunomodulatory treatment strategy in patients. Intervention studies in patients are ongoing and are expected to provide evidence for tailoring oxygenation in critically ill patients.

## Materials and Methods

### Effects of hyperoxia during murine endotoxemia

#### Animals

All procedures were in accordance with the requirements of the Dutch Experiments on Animals Act, the EC Directive 86/609 and approved by the Animal Ethics Committee of the Radboud University Medical Centre. A total of 32 male Balb/c mice (Charles River Laboratories International, Inc., L’Arbresle Cedex, France), aged six to eight weeks were used.

#### Reagents

Lipopolysaccharide (LPS, *E. Coli,* serotype 0111:B4) was obtained from Sigma–Aldrich (St Louis, MO, USA), dissolved in sterile 0.9% NaCl, and administered intraperitoneally at a dose of 5 mg/kg. Sterile 0.9% NaCl was used as placebo.

#### Experimental protocol

Mice were randomized into four groups (n = 8 per group); 1. normoxia-placebo. 2. normoxia-LPS. 3. hyperoxia-placebo. 4. hyperoxia-LPS. One hour before LPS/ placebo administration, mice were placed in an airtight cage with a continuous airflow of room air (21% oxygen, normoxia) or 100% oxygen (hyperoxia) to ensure a hyperoxic state during the initiation of inflammation. Ninety minutes after LPS/ placebo administration, when plasma levels of the archetypal pro-inflammatory cytokine TNFα (primary endpoint) reach their maximum^34^,^35^, mice were sacrificed by exsanguination through orbita extraction under deep isoflurane anesthesia. Blood was collected in ethylenediaminetetraacetic acid (EDTA)-containing tubes (MiniCollect, Greiner bio-one, Alphen a/d Rijn, the Netherlands), centrifuged (14000 g, 5 minutes, room temperature), and plasma was stored at −80°C until batch-wise analysis. Liver, spleen, and lungs were snap-frozen and stored at −80 °C until further analysis.

#### Cytokine analysis

Liver, spleen, and lung tissue was homogenized for 5 minutes at 50 Hz in Tissue Protein Extraction Reagent (Life Technologies, Bleiswijk, the Netherlands) containing protease inhibitors (complete EDTA-free tablets, Roche, Woerden, The Netherlands) using a Tissuelyzer LT instrument and 5 mm stainless steel beads (both from Qiagen, Venlo, the Netherlands Plasma and tissue homogenate concentrations of Tumor Necrosis Factor (TNF)-α, interleukin (IL)-6,keratinocyte-derived chemokine (KC, murine homologue of IL-8), and IL-10 were measured batch-wise using a Luminex assay (detection range 32–1000000 pg/mL) according to the manufacturer’s instructions (Milliplex, Merck Millipore, Billerica, MA, USA). Tissue cytokine concentrations were normalized to total protein content determined by bicinchoninic acid assay (BCA Protein Assay, Life Technologies, Bleiswijk, the Netherlands).

### Effects of hyperoxia during experimental human endotoxemia

#### Subjects and study design

Data was obtained from two larger trials on immunologic effects of oxygenation (ClinicalTrials.gov identifier NCT01889823 and NCT01978158). Experiments were in accordance with the declaration of Helsinki. After approval from the local ethics committee of the Radboud University Medical Center, 30 healthy male volunteers gave written informed consent to participate in the study. Subjects were screened before participation and had a normal physical examination, electrocardiography, and routine laboratory values. Subjects with febrile illness two weeks before the experiment, use of prescription drugs, or history of vagal collapse were excluded. The study was conducted in two phases. The first phase was designed to assess safety, feasibility, and immunologic effects of hyperoxia in the absence of systemic inflammation. In these experiments, 10 subjects were exposed to hyperoxia (fraction of inspired oxygen [FiO_2_] of 100%) using an air-tight respiratory helmet (CaStar, StarMed, Italy) for 3.5 hours. In the second phase, 20 subjects participated in endotoxemia experiments: they were randomized to either hyperoxia (as above, hyperoxic endotoxemia group, n = 10), or normoxia (FiO_2_ of 21% using the same respiratory helmet, normoxic endotoxemia group, n = 10). The period of hyperoxic exposure was chosen to assure a hyperoxic state at the initiation of the inflammatory response and throughout its acute phase, during which most inflammatory cytokines reach their maximum levels^36^,^37^. Apart from LPS administration, study protocols of the two phases were identical.

#### Study procedures

Experiments were conducted at the research unit of the intensive care department. Subjects refrained from caffeine and alcohol 24 hours before the experiment, and refrained from any intake of food and drinks 10 hours before the experiment. A venous and arterial cannula were placed for hydration, arterial blood pressure monitoring, and blood withdrawal. Heart rate was recorded with a three-lead electrocardiogram and peripheral saturation was measured using pulse oximetry. Hemodynamic data were recorded from a Philips MP50 patient monitor every 30 seconds using an in-house developed system. Every 30 minutes, temperature was measured using a tympanic thermometer (FirstTemp Genius 2; Covidien, Dublin, Ireland), and flu-like symptoms (headache, nausea, shivering, muscle and back pain) were scored on a six-point scale (0 = no symptoms, 5 = worst ever experienced), resulting in a total symptom score of 0–25.

After baseline measurements (t = −1 h), subjects were fitted with the respiratory helmet and normoxia or hyperoxia was initiated. The inflowing gas composition and the gas composition within the helmet were continuously monitored (%O_2_ and %CO_2_) using gas analyzers (Philips Airway Gases analyser M1026A, Philips Medical Systems, Boebligen, Germany) connected to patient monitors (Hewlett Packard Model M1166A 68S, Geneva, Switzerland and Capnomac Ultima, DATEX-OHMED, Helsinki, Finland). To prevent carbon dioxide build up, airflow was titrated to assure an inspiratory CO_2_ < 1%. One hour after initiation of normoxia or hyperoxia (t = 0 h), purified LPS (US Standard Reference Endotoxin Escherichia Coli O:113) obtained from the Pharmaceutical Development Section of the National Institutes of Health (Bethesda, MD, USA) was administered intravenously at a dose of 2 ng/kg in both endotoxemia groups. After 3.5 hours of normoxia or hyperoxia (t = 2.5 h), the helmet was removed and all subjects breathed room air for the rest of the experiment.

#### Blood gas parameters, lactate, and leukocyte counts

Blood gas parameters and lactate were analyzed in lithium heparin (LH)-anticoagulated arterial blood immediately after withdrawal using CG4+ cartridges and an i-STAT blood gas analyzer (Abbott, Libertyville, IL, USA). Analysis of leukocyte counts and differentiation was performed in EDTA-anticoagulated blood using routine patient sample analysis methods (flow cytometric analysis on a Sysmex XE-5000,The Netherlands).

#### Plasma cytokine concentrations and ex vivo whole bl*o*od stimulation

EDTA-anticoagulated blood was centrifuged immediately after withdrawal (2000 g, 10 minutes, 4 °C) and plasma was stored at −80 °C until analysis. “Concentrations of TNFα, IL-6, IL-8, and IL-10 were analyzed batch-wise using a Luminex assay (detection range 3.2-10000 pg/mL) according to the manufacturer’s instructions (Milliplex, Millipore, Billerica, MA, USA).” Leukocyte cytokine production capacity was assessed by *ex vivo* whole blood LPS stimulation using an in-house developed system^38^. Briefly, 0.5 mL LH-anticoagulated blood was added to pre-filled tubes with 2 mL culture medium or 2 mL culture medium supplemented with 12.5 ng/mL LPS (final LPS concentration: 10 ng/mL). After 24 hours of incubation at 37 °C, samples were centrifuged, and supernatants were stored at −80 °C until batch-wise analysis. Concentrations of TNFα and IL-6 were determined using ELISA according to the manufacturer’s instructions (Duoset, R&D systems, Minneapolis, MN, USA). Cytokine concentrations were normalized to monocyte counts (the main cytokine-producing cells in whole blood stimulation assays^39^).

#### Neutrophil phagocytosis assay

Neutrophil phagocytosis was measured using the pHrodo Red *E. coli* BioParticles Phagocytosis Kit for Flow Cytometry according to the manufacturer’s instructions (Life Technologies, Bleiswijk, the Netherlands). Briefly, 100 μL LH-anticoagulated blood was incubated with pHrodo Red *E. coli* BioParticles for 15 minutes (at 37 °C or on ice). Thereafter, erythrocytes were lysed, the leukocyte pellet was washed and stored in 1% paraformaldehyde solution. Analysis was performed using a Cytomics FC500 flow cytometer and Kaluza Analysis Software (both from Beckman Coulter; Galway, Ireland). Neutrophils were gated based on forward and side scatter characteristics. Using the samples incubated on ice as negative control, the percentage pHrodo-positive neutrophils in the phycoerythrin channel was determined. Phagocytic index was calculated as the percentage of pHrodo-positive neutrophils multiplied by the Mean fluorescence intensity (MFI) of the pHrodo-positive neutrophil population.

#### Neutrophil intracellular ROS generation

Neutrophil ROS generation was quantified using 2′,7′-dichlorofluorescein diacetate (DCFH-DA; Sigma, St Louis, MO, USA), as described previously^40^. Briefly, LH-anticoagulated blood was incubated with 0.06 mM DCFH-DA (30 minutes, 3 °C, 400 rpm), the reaction was stopped with 3 mM EDTA, and samples were centrifuged (430 g, 5 minutes, 4 °C). Erythrocytes were lysed (BD Pharm Lyse, Beckton Dickinson, Breda, the Netherlands) and cells were washed, after which the leukocyte pellet was resuspended in 3 mM EDTA solution and stored overnight at 4°, as pilot work showed that fluorescence remains stable overnight using this method. Analysis was performed the following day using a Cytomics FC500 flow cytometer and Kaluza Analysis Software (both from Beckman Coulter; Galway, Ireland). Pilot studies have shown that the fluorescence remains stable when stored overNeutrophils were gated based on forward and side scatter characteristics, and ROS generation was quantified by determining the mean fluorescence of the neutrophil population. ROS generation was normalized to baseline levels (t = −1 h).

#### Calculations and statistical analysis

Data are expressed as median [interquartile range] or mean ± SEM based on their distribution (calculated by Shapiro-Wilk tests). Except for demographic characteristics, all non-parametric data were log-transformed before statistical analysis. Differences between groups in the murine experiments and between-group differences in mean PaO_2_, peak levels of flu-like symptoms, and demographic characteristics in humans were evaluated using unpaired Student’s t-tests or Kruskall-Wallis tests. Within-group differences over time in the hyperoxia group were analyzed using one-way analysis of variance (ANOVA). Two-way ANOVA (interaction term) was used to assess differences between endotoxemic groups over time. A p-value of < 0.05 was considered statistically significant. Statistical calculations were performed using GraphPad Prism version 5.03 (GraphPad Software, San Diego, CA, USA).

## Additional Information

**How to cite this article**: Kiers, D. *et al.* Short-term hyperoxia does not exert immunologic effects during experimental murine and human endotoxemia. *Sci. Rep.* **5**, 17441; doi: 10.1038/srep17441 (2015).

## Figures and Tables

**Table 1:** Demographic characteristics.

	HYPER	NORM-E	HYPER-E	p
Age [yr)	23 [20–24]	21 [20–23]	21 [20–24]	0.68
Height [cm)	182 [178–184]	185 [180–192]	182 [179–189]	0.60
Weight [kg)	76 [74–84]	75 [69–86]	72 [67–80]	0.61
BMI [kg/m^2^)	23.8 [22.8-24.8]	22.2 [20.5-24.2]	22.5 [18.8-24.3]	0.28

Data obtained during screening visit and presented as median [interquartile range]. NORM-E: normoxic endotoxemia, HYPER-E: hyperoxic endotoxemia, HYPER: hyperoxia, yr: years; cm: centimeter, kg: kilogram, BMI: body mass index, m: meter.
